# Synovial calprotectin in prosthetic joint infection. A systematic review and meta-analysis of the literature

**DOI:** 10.1007/s00402-024-05416-0

**Published:** 2024-07-08

**Authors:** E. Festa, T. Ascione, D. Di Gennaro, D. De Mauro, M. Mariconda, G. Balato

**Affiliations:** 1 Department of Public Health, Orthopedic Unit, “Federico II” University, Naples, Italy; 2 Service of Infectious Diseases, Cardarelli Hospital, Naples, Italy; 3 Department of Orthopedics and Geriatric Sciences, Catholic University of Sacred Heart, Rome, Italy

**Keywords:** Calprotectin, Prosthetic joint infection, Hip, Knee

## Abstract

**Introduction:**

Calprotectin is a protein endowed with antimicrobial properties, rendering it a distinctive marker for infection. Two methods are currently available for the assay of calprotectin: the enzyme-linked immunosorbent assay (ELISA) and the lateral flow test (LFT). We aimed to assess the diagnostic accuracy of synovial fluid calprotectin and to compare the accuracy of the laboratory-based test and the qualitative assessment for the diagnosis of hip and knee prosthetic infection.

**Materials and methods:**

We searched (from inception to November 2023) MEDLINE, Scopus, EMBASE, Web of Science, and Cochrane for studies on calprotectin in the diagnosis of periprosthetic joint infection (PJI). Sensitivity, specificity, positive and negative likelihood ratio (LR), and diagnostic odds ratio were analyzed. The receiver-operating curve for each method was calculated.

**Results:**

We included 14 articles in our meta-analysis, including 902 patients who underwent total hip and knee arthroplasties revision; 331 (37%) had a joint infection according to MSIS, MSIS-modified criteria, ICM 2018 and EBJIS 2021. Considering the false-positive result rate of 6% and false-negative result rate of 7%, pooled sensitivity and specificity were 0.92 (95% CI 0.89–0.94) and 0.93 (0.91–0.95), respectively. The area under the curve (AUC) was 0.93 (95% CI 0.91–0.94). No statistical differences in terms of sensitivity and specificity were found between ELISA and LFT. The pooled sensitivity and specificity of the two calprotectin assessment methods were: LFT 0.90 (95% CI 0.869–0.935) and 0.92 (95% CI 0.894–0.941), respectively; ELISA 0.96 (95% CI 0.914–0.986) and 0.97 (95% CI 0.934–0.988), respectively. The diagnostic odds ratio of the ELISA was superior to that of the LFT (906.6667, 95% CI 271.2686–3030.3712 versus 113.8886, 95% CI 70.4001-184.2414; *p* < 0.001). The AUC for ELISA and LFT was 0.968 (95% CI 0.944–0.984) and 0.915 (95% CI 0.895–0.933), respectively.

**Conclusions:**

Detection of synovial calprotectin is an accurate test for diagnosis of hip and knee prosthetic infections. The diagnostic accuracy of the two calprotectin assessment methods is almost comparable. The LFT is a valid, rapid, and more available diagnostic tool, particularly to rule out PJI.

**Supplementary Information:**

The online version contains supplementary material available at 10.1007/s00402-024-05416-0.

## Introduction

Periprosthetic joint infection (PJI) is one of the most serious complications after total hip arthroplasty (THA) and total knee arthroplasty (TKA). It is currently the most common indication for early revision TKA and the second indication for late revision TKA (31.3% and 22.2% respectively) and the fourth most common indication for revision total hip arthroplasty (16% of all hip revisions) worldwide [1–4]. Although a definite preoperative diagnosis of septic failure is imperative for proper treatment and management, the diagnosis of PJI remains a serious clinical challenge [5–8]. Unfortunately, no gold standard exists, and no single test is available with 100% diagnostic accuracy to detect an infection. Serological markers such as CRP, D-dimer, and ESR have been widely used in diagnosing PJI; they are highly influenced by various systemic and confounding factors [9–11]. The emergence of new diagnostic modalities has made synovial biomarkers of particular interest, including synovial WBC, leukocyte esterase, and Alpha-Defensin [11–14], which have shown promising potential as diagnostic tools in PJI. Since then, other synovial biomarkers have been investigated. Among them, synovial calprotectin, secreted by neutrophilic granulocytes and monocytes at sites of local inflammation, plays a role in leukocyte migration and stimulation [15–31], thus making calprotectin an intriguing biomarker for PJI [32]. There are two available methods for measuring calprotectin in synovial fluid. The calprotectin ELISA Immunoassay is based on colorimetric detection using monoclonal and polyclonal antibodies against calprotectin, while the calprotectin lateral flow test (LFT) is a quantitative detection of synovial calprotectin, which has the advantage of immediate availability of results, so it is useful for intra-operative diagnosis of PJI. Recently, three meta-analyses analyzed the accuracy of this marker for the diagnosis of PJI and concluded that calprotectin has an excellent diagnostic accuracy [29–31]. Since then, further published studies have evaluated the accuracies of the LFT and the ELISA one, so a meta-analysis that includes these emerging studies is needed to verify the accuracy of the previous results and to compare the two methods. Furthermore, we designed the present meta-analysis because the available evidence on these two tests has not been investigated exclusively on hip and knee prosthetic infections.

We therefore asked: (1) What is the role of synovial fluid calprotectin as a biomarker for infection of joint prostheses? (2) What is its reliability and validity in terms of sensitivity, specificity, diagnostic odds ratio (DOR), positive predictive value, negative predictive value, and area under the curve (AUC)? (3) Which method, the LFT or ELISA, offers more advantages?

## Materials and methods

### Calprotectin assessment methods

Two calprotectin assessment methods are available. For the calprotectin LFT, 20 µl of each joint fluid aspirate was added to 2 ml of dilution buffer. Subsequently, 80 µl of the mix was pipetted onto a well in the test cartridge. Calprotectin is bound by a specific antibody complex on the membrane, resulting in a visible test line for colorimetric detection. The remaining antibody complexes flow laterally and are immobilized on a control line. The color intensity of the test line is proportional to the calprotectin concentration. After 15 min, the test results were photometrically assessed using a smartphone application provided by Lyfstone. Three categories were defined when measuring the calprotectin concentration: < 14 mg/ml or low risk, 14–50 mg/ml or moderate risk, and 50– > 300 mg/ml or high risk for infection [26]. In the laboratory-based test, aliquots for calprotectin testing were subjected to centrifugation. The immunoassay for synovial fluid calprotectin was generated using monoclonal and polyclonal antibodies against calprotectin adsorbed to the surface of plastic wells [17]. Most studies have used 50 mg/L as the threshold value [15–17, 21–23].

### Data sources and search strategy

We searched for studies investigating diagnostic accuracy of synovial fluid calprotectin in patients with periprosthetic infections in the MEDLINE, Scopus, EMBASE, Web of Science, and Cochrane databases from inception to November 2023. The Preferred Reporting Items for Systematic Review and Meta-Analyses (PRISMA) methodology guidance was employed [33]. The search strategy used a combination of the following key words: calprotectin AND PJI OR periprosthetic joint infection OR prosthetic infection, with no language restrictions. The reference lists of selected articles were also hand-searched for any additional articles that were not identified from the database search.

### Eligibility criteria

Longitudinal studies (retrospective and prospective) and randomized controlled trials evaluating the diagnostic accuracy of these ratios in PJI were finally selected. Studies evaluated the diagnostic accuracy of synovial fluid calprotectin measured either by the immunoassay and lateral flow test in the diagnosis of PJI were included. Papers considered MSIS or later modified MSIS criteria, or ICM 2018, or EBJIS 2021 as reference standard for the diagnosis of PJI were also included. The exclusion criteria included: case reports, expert opinions, previous metanalysis and systematic reviews, letters to the editor, studies that did not report quantitative values of sensitivity, specificity or likelihood ratios, or diagnostic accuracy.

### Study assessment and data extraction

Initial screening of titles and abstracts was performed by two pairs of independent reviewers. The full text was obtained for all abstracts that appeared to meet the inclusion criteria or where there was any uncertainty. Each study was assessed by two independent reviewers using the inclusion criteria and any discrepancies regarding the eligibility of an article were resolved with a third author. Relevant data were extracted from each included study. Two authors performed quality assessment of each study using the QUADAS (Quality Assessment of Diagnostic Accuracy Studies) tool [34]. The QUADAS score consists of four domains: (1) patient selection, (2) index test, reference standard, (3) flow, and (4) timing. The risk of bias assessment of the four domains and the clinical applicability of the first three domains were assessed with signaling questions. Questions were answered “yes” for low risk of bias/concerns, “no” for high risk of bias/concerns, or “unclear”.

## Results

**Fig. 1:**
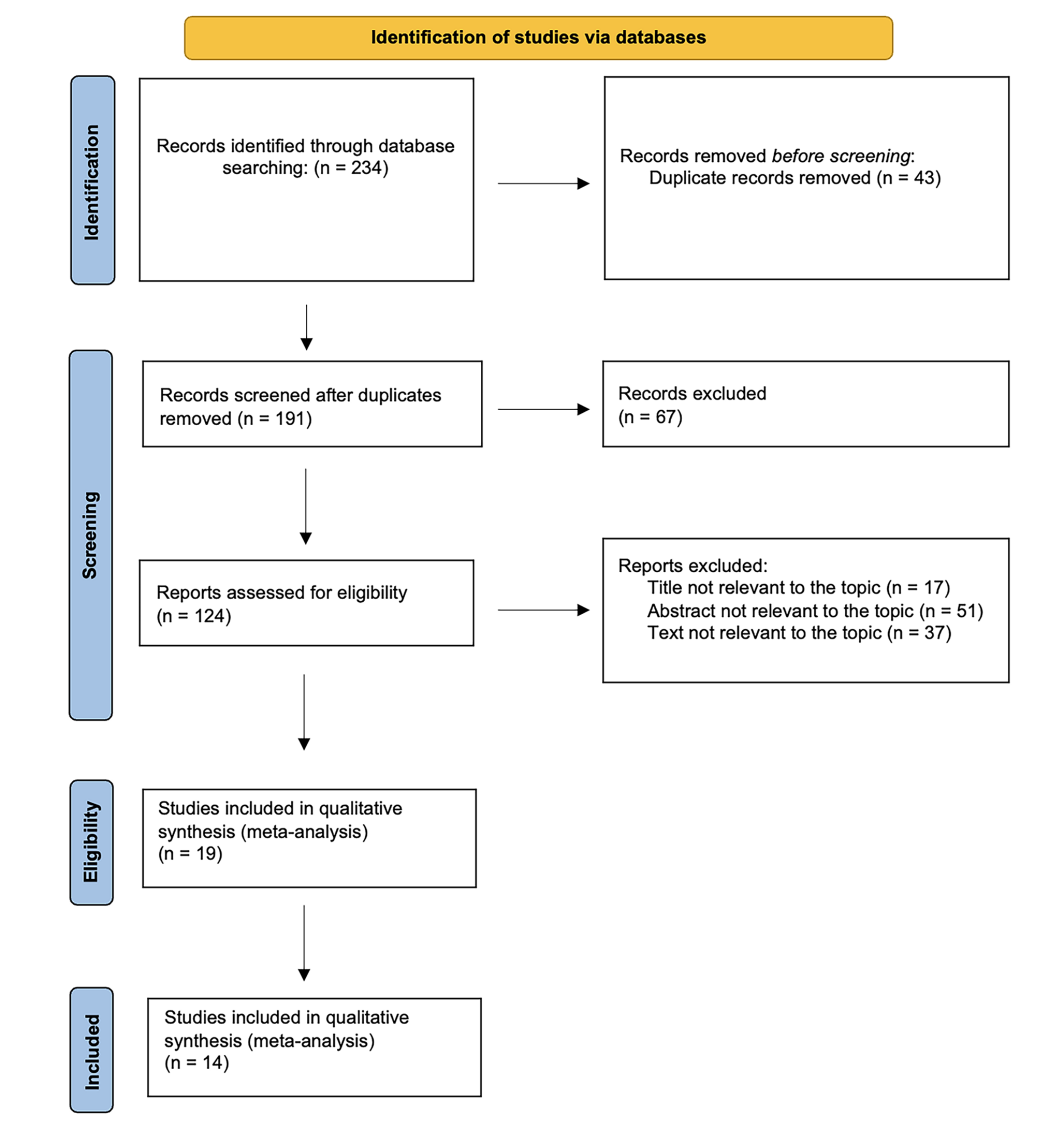
PRISMA flow diagram

**Fig. 2:**
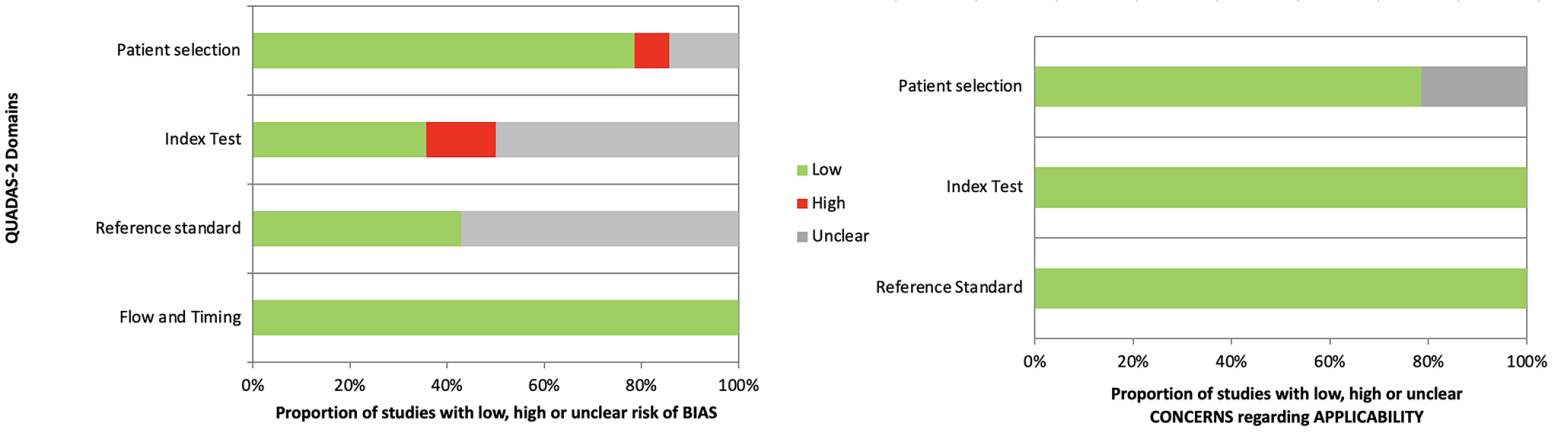
QUADAS-2 scores for studies included in the meta-analysis

The flow diagram of our search strategy is reported in Fig. 1. Computer search and manual screening of reference lists of relevant studies identified 234 potentially relevant citations. After initial screening of titles and abstracts, the full text of 124 articles was evaluated. After detailed assessment, we excluded 110 references. The remaining 14 articles were included in our meta-analysis. Table 1 summarizes the characteristics of the included studies. In total, 902 patients who underwent total joint arthroplasties revision were evaluated, among whom 331 (37%; range 22–53%) were confirmed to have a joint infection according to MSIS, MSIS-modified criteria., ICM 2018 and EBJIS 2021. The number of joints included was explained in all papers and the site of infection was described in all paper but one [28]. The sensitivity, specificity, positive and negative likelihood ratio, and DOR of included studies, and their corresponding pooled indices, are shown in Table 2. The area under the curve (AUC) was 0.93 (95% CI 0.91–0.94). The results of the QUADAS-2 are in Fig. 2.

**Table 1:** Characteristics of included studies

Lead Author	Study design	No.^a^ Patients (PJI%)	Age	Detection Method	Reference standard	Site of arthroplasty in infected group Hip/knee/shoulder/ankle
Wouthuyzen-Bakker et al. [15]	Prospective	19/61 (31%)	Septic 65 (24–87) Aseptic 60 (23–90)	LFT	MSIS 2011	11/5/3/0
Wouthuyzen-Bakker et al. [16]	Prospective	15/52 (29%)	NA	LFT	MSIS 2011	8/5/2/0
Salari et al. [17]	Prospective	28/72 (39%)	69	ELISA	ICM 2018	0/28/0/0
Zhang et al. [18]	Prospective	21/63 (33%)	Septic 64 (54–83) Aseptic 57 (41–86)	ELISA	MSIS 2013	15/6/0/0
Trotter et al. [19]	Retrospective	24/69 (35%)	74.3 (45–89)	LFT	ICM 2018	15/9/0/0
Grzelecki et al. [20]	Prospective	45/85 (53%)	Septic 65.5 ± 10 Aseptic 68.3 ± 12	LFT	ICM 2018	20/25/0/0
Warren et al. [21]	Prospective	53/123 (43%)	Septic 66.9 ± 10.6 Aseptic 65.4 ± 10.6	LFT + ELISA	MSIS 2013	0/53/0/0
Grassi et al. [22]	Prospective	39/89 (44%)	77	LFT + ELISA	ICM 2018	0/39/0/0
Warren et al. [23]	Prospective	53/123 (43%)	Septic 66.9 ± 10.6 Aseptic 65.4 ± 10.6	LFT	MSIS 2013 ICM 2018 Proposed EBJIS 2019	0/53/0/0
Lazic I. et al. [24]	Prospective	17/33 (51%)	65.1 ± 13.9	LFT	EBJIS 2021	13/4/0/0
Lazic I. et al. [25]	Prospective	14/30 (46%)	Septic 71.8 ± 14 Aseptic 72.7 ± 15.1	LFT	EBJIS 2021	10/4/0/0
Suren et al. [26]	Prospective	34/137 (25%)	Septic 70 ± 11 Aseptic 67 ± 13	LFT	ICM 2018	16/18/0/0
Lazic et al. [27]	Prospective	10/33 (30%)	73.3 ± 11.4	LFT	EBJIS 2021	6/4/0/0
Bottagisio et al. [28]	Prospective	12/55 (22%)	Septic 70 ± 15 Aseptic 72 ± 10	LFT	MSIS 2013 ICM 2018	-

^a^The values were given as the number of PJI/total of joint involved in study

EBJIS: European Bone and Joint Infection Society; ELISA: enzyme-linked immunosorbent assay; ICM: International Consensus Meeting; LFT: Lateral Flow Test; MSIS: Musculoskeletal Infection Society

**Table 2:** Characteristics of diagnostic studies for calprotectin

Lead Author	Optimal cutoff	Sensitivity	Specificity	AUC	LR+	LR-	PPV	NPV	DOR (IC 95%)	Mean calprotectin concentration in septic Vs. aseptic joints
Wouthuyzen-Bakker et al. [15]	> = 50 mg/L	89%	90%	0.94	8.9	0.1	81%	95%	80.7500 (13.4663–484.2120)	991 mg/L Vs 11 mg/L
Wouthuyzen-Bakker et al. [16]	> = 50 mg/L	86.7%	91.7%	0.94	10.9	0.14	81.3%	94.4%	73.6667 (11.0187-492.5054)	859 mg/L Vs 7 mg/L
Salari et al. [17]	> = 50 mg/L	100%	95%	0.996	22	0	-93.33%	100%	969.0000 (44.8350-20942.5933)	320 mg/L Vs 5.5 mg/L
Zhang et al. [18]	173 µg/ml	95.2%	97.6%	0.993	39.6	0.049	95.2%	97.6%	820.0000 (48.7335-13797.4917)	776 µg/ml Vs 54.5 µg/ml
Trotter et al. [19]	> = 14 mg/L	75%	75.56%	0.78	3.07	0.33	62.07%	85%	9.2727 (2.9445–29.2012)	-
Grzelecki et al. [20]	1.5 mg/L	95.6%	95%	0.95	19.11	0.05	95.5%	95%	408.5000 (54.8444-3042.6512)	20.46 mg/L Vs 0.7 mg/L
Warren et al. [21]	* > = 50 mg/L > = 14 mg/L	ELISA 98.1% POC 98.1%	ELISA 95.7% POC 95.7%	ELISA 0.969 POC 0.969	ELISA 22.89 POC 22.89	ELISA 0.02 POC 0.02	ELISA 94.5% POC 94.5%	ELISA 98.5% POC 98.5%	ELISA and POC 1161.3333 (117.3670-11491.2629)	-
Grassi et al. [22]	> = 50 mg/L	ELISA 92.3% POC 97.4%	ELISA 100% POC 94%	ELISA 0.962 POC 0.957	ELISA NA POC 16.239	ELISA 0.077 POC 0.027	ELISA 100% POC 92.7%	ELISA 94.3% POC 97.9%	ELISA 1053.2857 (52.7725-21022.5283) POC 595.333 (59.4937-5957.2978)	ELISA 290.6 mg/L Vs 6.5 mg/ L
Warren et al. [23]	> = 50 mg/L	^a^ 98.2%	98.5%	0.984	-68.68	0.02	98.2%	98.5%	3588.0000 (219.2516-58716.7506)	-
Lazic I. et al. [24]	70.5 mg/ dl	88%	88%	0.89	7.06	0.13	88.24%	87.5%	52.5000 (6.4874–424.8650)	-
Lazic I. et al. [25]	76 mg/dl	71%	81%	0.77	3.81	0.35	77%	76%	10.8333 (1.9614–59.8358)	-
Suren et al. [26]	85.5 mg/L	92%	95%	0.94	23.48	0.09	88.57%	97.06%	255.7500 (54.2621-1205.4094)	-
Lazic et al. [27]	70 mg/dl	60%	78%	0.71	2.76	0.51	54.55%	81.82%	5.4000 (1.0826–26.9339)	-
Bottagisio et al. [28]	529.5 µg/g	80%	94.4%	0.852	11.7	0.179	-	-	40.0000 (6.9086-231.5967)	874 µg/g Vs 30 µg/g
Pooled		92.44%	93.43%	93.05	14.07	0.08	89.8%	95.18%		

*Two cutoffs were used, but only the > = 50 mg/L was used in the analysis

^a^ Three system criteria were analyzed, but only the ICM 2018 was included in the analysis

AUC: Area Under the Curve; LR+: positive likelihood ratio; LR-: negative likelihood ratio; POC: point of care; PPV: positive predictive value; NPV: negative predictive value

### Diagnostic accuracy of LFT vs. ELISA of calprotectin in synovial fluid

**Fig. 3:**
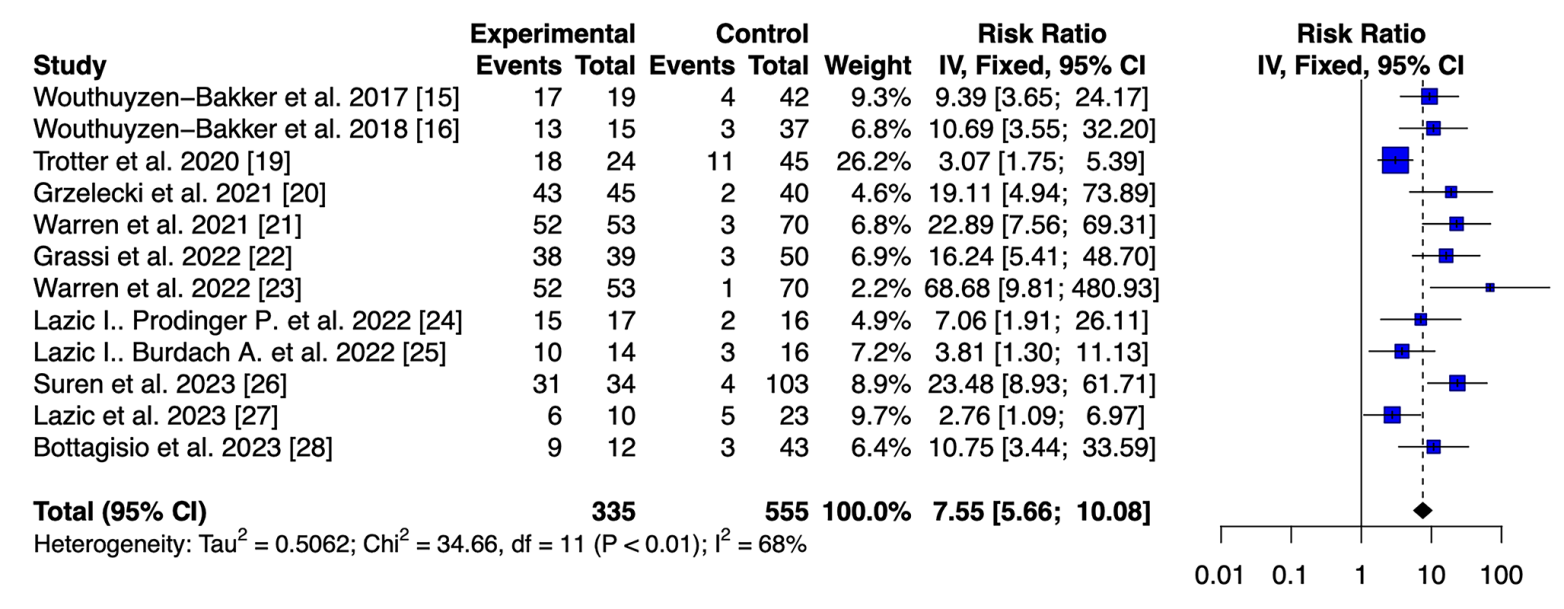
Calprotectin POC Test: A forest plot showing the study-specific and meta-analyzed estimates for risk ratio utilizing the Fixed-effect model

**Fig. 4:**
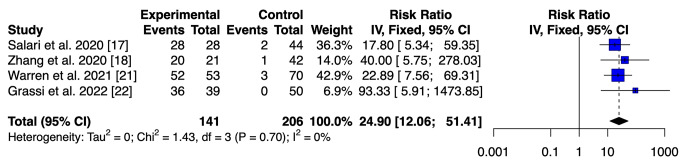
Calprotectin ELISA: A forest plot showing the study-specific and meta-analyzed estimates for risk ratio utilizing the Fixed-effect model

The pooled sensitivity of the LFT and ELISA was 0.90 (95% CI 0.869–0.935) and 0.96 (95% CI 0.914–0.986), respectively. The pooled specificity for LFT was 0.92 (95% CI 0.894–0.941). Better results in term of specificity were reported for ELISA with a value of 0.97 (95% CI 0.934–0.988). No statistical differences in terms of sensitivity and specificity were found between the two assays. The ELISA had a superior DOR value to that of the LFT (906.6667, 95% CI 271.2686–3030.3712 versus 113.8886, 95% CI 70.4001-184.2414; *p* < 0.001). Furthermore, the ELISA had a higher positive likelihood ratio 33.115 (95% IC 15.044–72.897) versus 11.446 (95% IC 8.602–15.229. A lower value of negative LR was retrieved for the ELISA 0.036 (95% IC 0.015–0.086) versus 0.100 (95% IC 0.071–0.14). In addition, the AUC for the LFT was 0.915 (95% CI 0.895–0.933), whereas the AUC for the ELISA was 0.968 (95% CI 0.944–0.984). Utilizing the DeLong’s test, we found a statistical difference between the two accuracy values (*p* < 0.005) [35]. Furthermore, we used the fixed-effect model for both tests [36]. The analysis obtained a risk ratio (RR) of 7.55 (95% IC 5.66–10.08) and 24.90 (95% IC 12.06–51.41), respectively (Figs. 3 and 4).

## Discussion

The main findings of this study are that calprotectin shows high diagnostic accuracy in the diagnostic workup of painful total joint arthroplasty independently from the assessment methods. This would position it among the emerging biomarkers for diagnosing PJIs, alongside fibrinogen and others [37, 38]. Recent meta-analyses reported the high diagnostic accuracy of synovial calprotectin [29–31]. Although further studies have been published, the pooled estimation of the 14 studies included in our study indicates a similar specificity and sensitivity rate result. Considering the false-positive result rate of 11% (50/440) and false-negative result rate of 5% (36/711), the pooled sensitivity and specificity were 0.924 (95% CI 0.895–0.945) and 0.934 (95% CI 0.913–0.950), respectively. The studies included did not clarify why patients with joint infection report average levels of calprotectin in synovial fluid. Bloody and clotted aspirates could explain negative calprotectin results, as described in different papers [24]. Another possible reason could be attributed to low-grade infections, generally sustained by less virulent bacteria such as *P. acnes* or coagulase-negative Staphylococci, which are a condition where clinical and laboratory criteria may misdiagnose PJI [39, 40]. Micro-organisms of low virulence rapidly adhere to implants, evading host defense and resulting in a weak immune response [39–41]. The dampened inflammation could explain the increase of false-negative results of serum and synovial fluid biomarkers in low-grade PJI. Antibiotic treatment does not appear to give false-negative results [42, 43]. Zhang et al. analyzed the concentration of calprotectin in two groups. In the antibiotic treatment group, this marker was 663 (IQR, 480 to 1,106) µg/ml, while in the non-antibiotic treatment group was 792 (IQR, 577 to 1,203) µg/ml. This difference was not statistically significant (*p* = 0.343). However, regardless of the use of antibiotics, the concentration of calprotectin in the PJI group was significantly higher than that in the aseptic failure group, and the difference was statistically significant (*p* < 0.001) [18].

Regarding the false positives, the ability of calprotectin to role in PJI can be modified by inflammatory non-infectious conditions [28] or severe osteolysis or the metallosis caused by metal-to-metal reactions [19]. However, Suren, in his paper, reported that patients with metallosis showed calprotectin levels < 14 mg/l, suggesting that the presence of inflammation caused by metallosis might not induce false-positive calprotectin levels [26]. On the other hand, he confirmed that the calprotectin levels could increase in patients with marked osteolysis and wear disease. There is the inflammatory foreign-body reaction to debris particles due to the activation of monocytes and macrophages instead of the neutrophil activation being more predominant in bacterial infection. The positive and negative likelihood ratios of calprotectin were 14.068 (95% CI 10.749–18.414) and 0.080 (95% CI 0.059–0.110), respectively. This finding demonstrated that a positive or negative result for calprotectin indicates an increased or decreased probability of PJI. Moreover, the DOR and AUC in our study support this finding. In our analysis, calprotectin had a high diagnostic utility with elevated discriminatory test performance between patients with and without a PJI, as demonstrated by a DOR of > 1 and an AUC of 0.93 (95% CI 0.91–0.94).

Furthermore, we divided the studies into two groups to distinguish the results between those obtained with LFT test and those with ELISA. We compared the diagnostic accuracy of the ELISA and LFT tests to diagnose TKA and THA infections. Both assays had high diagnostic accuracy, but the analysis of synovial calprotectin with ELISA reported higher diagnostic indices for diagnosing PJI. Even lower results in terms of the pooled sensitivity and specificity were found for the qualitative test. There was a statistical difference between the two accuracy values.

**Fig. 5:**
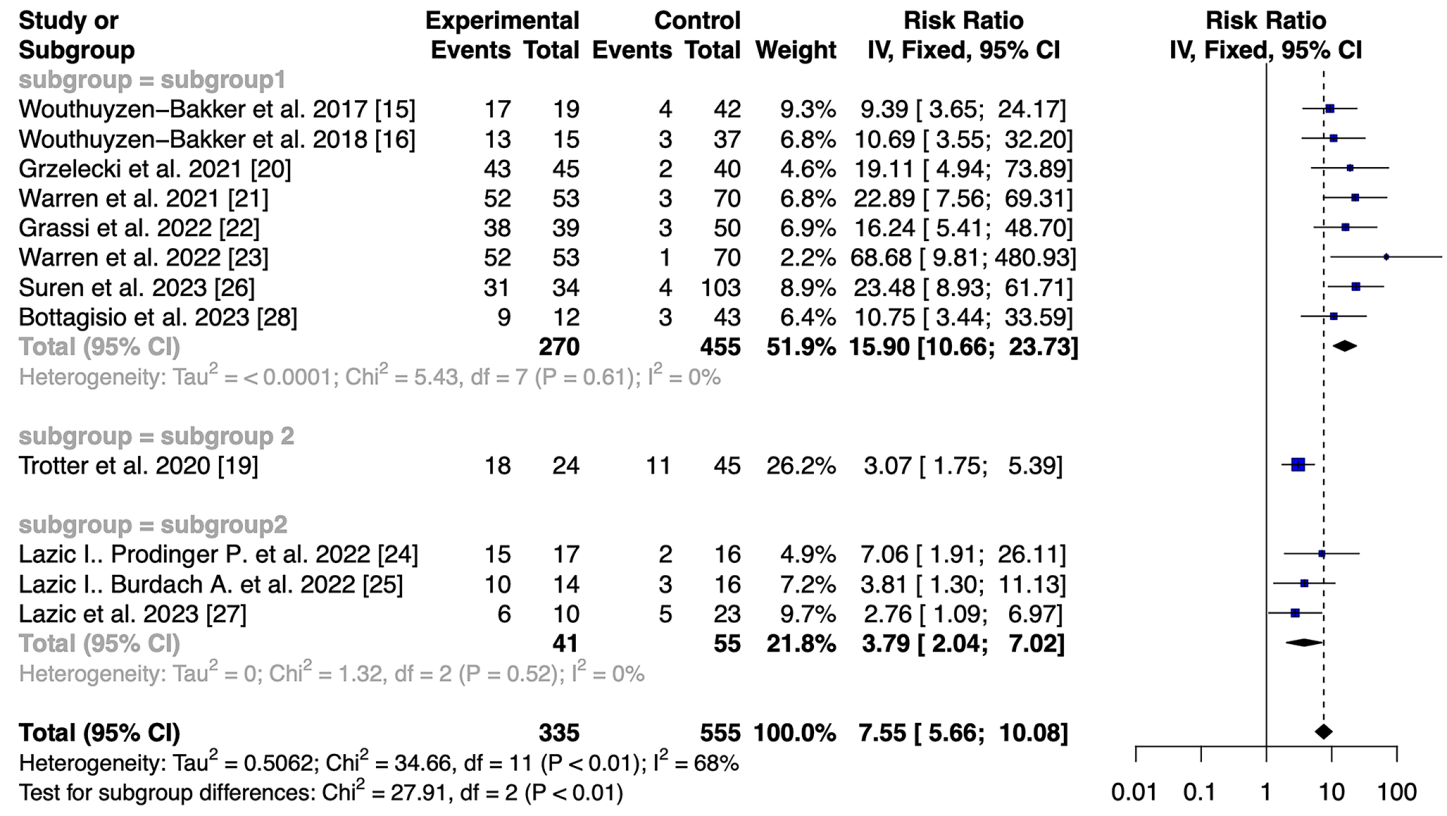
Subgroup analysis utilizing the Fixed-effect model

We utilized the fixed-effect model for both tests because we assumed that the only source of variability between the results obtained from the studies is the different sampling that characterizes them. Any differences in observed effects are due to sampling error. Regarding the ELISA, there is no heterogeneity in the studies we pooled (I2 = 0%; *p* = 0.70), with an overall risk ratio of 24.90 (95% CI 12.06–51.41). For the LFT, there is a degree of heterogeneity in the studies we considered (I2 = 68%; *p* = < 0.01). Therefore, we performed a subgroup analysis to find the source of heterogeneity. The results of the subgroup analyses suggested that the heterogeneity may result from two main elements: the type of study and the threshold that has been utilized. The heterogeneity is annulled when we proceed to remove the only retrospective study by Trotter et al. [19] and those by Lazic et al., who utilized a different threshold for synovial calprotectin [24, 25, 27] (Fig. 5).

The strengths and potential limitations of this study should be acknowledged. This study is the first meta-analysis on the utility of calprotectin in hip and knee prosthetic infections. We adopted stringent eligibility criteria that led to the exclusion of studies that assessed calprotectin results in patients with PJI that differed from TKA and THA. Studies that reported infections of other joints were excluded only if the specific data (TKA and THA PJI) could not be extrapolated. Another strength of this study is the increased number of included studies on lateral flow test and ELISA compared with that of previous metanalyses. We analyzed fourteen papers in contrast to the 8 of Hantouly et al. [29], 7 of Peng et al. [30], and 7 of Xing et al. [31], respectively.

This study has a few drawbacks. First, the different diagnostic criteria used to rule out PJI and the small number of patients included in the studies may have contributed to the heterogeneity among studies that emerged for some outcomes assessed in the present meta-analysis. Furthermore, many studies do not include in their diagnostic workup for PJI some criteria included in MSIS and modified MSIS criteria. This disparity could alter the ability of these diagnostic criteria to distinguish between septic and aseptic loosening and, consequently, change the accuracy of new diagnostic tests. Lastly, some confounders, such as chronic inflammatory disease, metallosis of patients included, and use of concomitant antibiotic treatment, may be responsible for false results of the calprotectin evaluation and represent another limitation of this study. We know that rheumatic disease is one of the most important reasons for false-positive results, as is the presence of metallosis.

## Conclusion

Detection of synovial calprotectin is an accurate test that helps to diagnose hip and knee prosthetic infections. The diagnostic accuracy of the two calprotectin assessment methods analyzed is comparable. Because the results are available within 15 min with the LFT, this test is a valuable and accurate addition to the pre-operative diagnostic workup before arthroplasty exchange, especially in cases where the gold standard results are inconclusive and particularly when we want to rule out the PJI.

## Electronic supplementary material

Below is the link to the electronic supplementary material.

Supplementary Material 1
